# The Red-Colored Oddball—A New Ladybird Spider with Unusual Coloring from Morocco, *Eresus rubrocephalus* sp. nov. (Araneae: Eresidae)

**DOI:** 10.3390/ani15182707

**Published:** 2025-09-16

**Authors:** János Gál, Gábor Kovács, Zoltán Vincze, Gergő Keve, Barna Páll-Gergely, Richárd Bagyó, Enikő Fehér, Krisztina Bali, Eszter Kaszab

**Affiliations:** 1 Department of Exotic Animal, Wildlife, Fish and Honeybee Medicine, University of Veterinary Medicine Budapest, H-1078 Budapest, Hungary; 2 College of Veterinary Medicine, Huazhong Agricultural University, Wuhan 430070, China; 3 Independent researcher, Londoni körút 1, H-6724 Szeged, Hungary; 4 Department of Parasitology and Zoology, University of Veterinary Medicine Budapest, H-1078 Budapest, Hungary; 5 Department of Zoology, Plant Protection Institute, HUN-REN Centre for Agricultural Research, H-2462 Martonvásár, Hungary; 6 Independent researcher, Rue Melouiya, Agdal Ryad 60, Rabat 10000, Morocco; 7 Department of Microbiology and Infectious Diseases, University of Veterinary Medicine Budapest, H-1078 Budapest, Hungary; 8 National Laboratory for Infectious Animal Diseases, Antimicrobial Resistance, Veterinary Public Health and Food Chain Safety, H-1078 Budapest, Hungary; 9 National Laboratory of Virology, Szentágothai Research Centre, University of Pécs, H-7624 Pécs, Hungary; 10 Department of Bioinformatics, One Health Institute, Faculty of Health Sciences, University of Debrecen, H-4032 Debrecen, Hungary

**Keywords:** velvet spiders, North Africa, genetic analysis, COI analysis, species delimitation

## Abstract

According to our current knowledge, the prothorax of male spiders belonging to the genus *Eresus* is covered with black hairs. However, during our collection activities in Morocco, we found male specimens showing habitus that can be clearly distinguished from the previously known species based on their pars cephalica of prosoma covered with distinct red hairs. Diagnostic drawings and digital photographs of male copulatory organs, alongside DNA and COI barcoding results, are also presented.

## Simple Summary

In our work, we provide a description of the habitus of a species of ladybird spider found in Northern Africa, specifically Morocco, based on microscopic examination of the palpus and genetic delimitation analysis. The cephalothorax and abdomen of the male spider are both covered with carmine red hairs on the dorsal and ventral sides as well as on the chelicerae. The palpus exhibits several characteristic distinguishing features, such as the course of the palpus conductor plate, the characteristic U-shaped groove, and the uniquely shaped terminal tooth. Both phylogenetic and species delimitation analyses supported the establishment of the new species *Eresus rubrocephalus* sp. n.

## 1. Introduction

Of the nine genera in the family Eresidae C. L. Koch, 1845 (“velvet spiders”), the genus *Eresus* Walckenaer, 1805 (“ladybird spiders”) has thirty-eight species; furthermore, *Eresus kollari* Rossi, 1846, has four (*E. k. frontalis* Latreille, 1817; *E. k. ignicomis* Simon, 1914; *E. k. latefasciatus* Simon, 1911; *E. k. tricolor* Simon, 1873), and *Eresus walckenaeri* Brullé, 1832, has one (*E. w. moerens* C. L. Koch, 1846) additional subspecies. Most species of the family are found in North Africa, Central and Southern Europe, the Middle East, and Asia [1,2,3,4,5,6,7,8,9,10,11,12,13]. From the northwestern part of the African continent, Morocco, the occurrence of *Eresus almaghrib* Szűts, Lecigne & Moutaoualkil, 2025, *Eresus gharbi* Szűts, Lecigne & Moutaoualkil, 2025, and *Eresus elhennawyi* Rezác, Vanek & Strestík, 2023, was described, but the latter has also been found in neighboring Algeria [14]. The authoritative male specimen of *E. gharbi* is listed in the literature as having been collected in Sidi Ifni, Morocco, while a male specimen of *E. almaghrib* is listed as having been collected in Essaouira [15]. The occurrence of *E. elhennawyi* in Morocco is reported in Agadir, Taroudant, and Guelmin, while in Algeria it is reported in the city of Laghouat [16].

**Figure 1:**
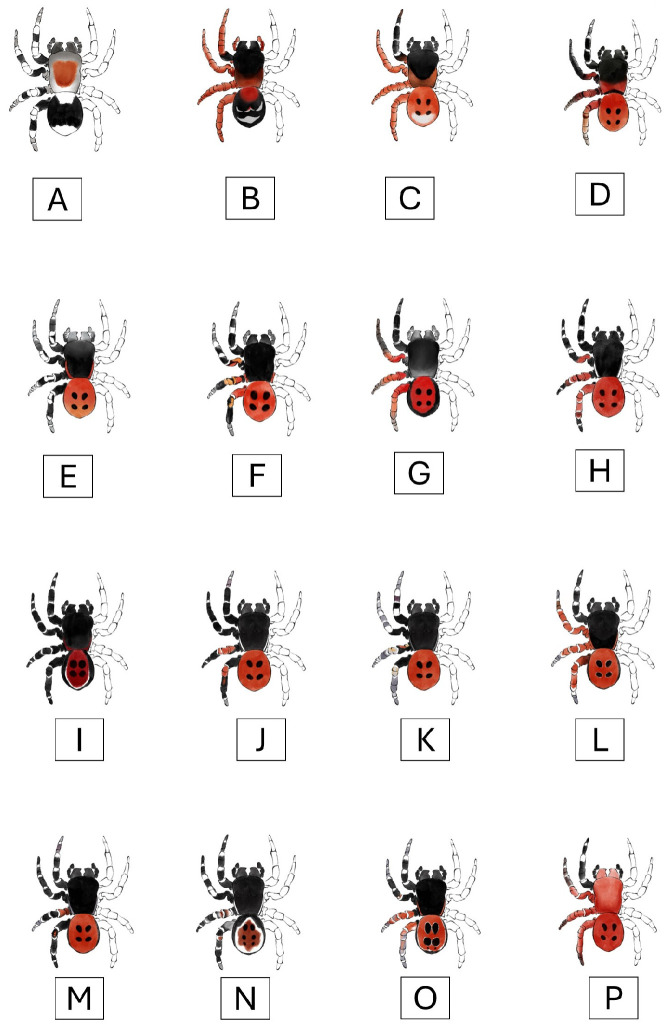
Habitus drawing of a male *Eresus* species. (**A**) *E. elhennawyi*, (**B**) *E. almaghrib*, (**C**) *E. gharbi*, (**D**) *E. moravicus*, (**E**) *E. sandaliatus*, (**F**) *E. kollari*, (**G**) *E. hermani*, (**H**) *E. urus*, (**I**) *E. granosus*, (**J**) *E. surena*, (**K**) *E. rezaci*, (**L**) *E. agrinus*, (**M**) *E. robin*, (**N**) *E. athanatoi*, (**O**) *E*. *transcaucasicus,* and (**P**) *E. rubrocephalus* sp. nov. Drawn by Anna Ditta Dénes based on the work of Lecigne et al., 2025 [15], Rezác et al., 2023 [16], Zamani et al., 2025 [13], Kovács et al., 2015 [17], Al-Yacoub et al., 2025 [21], and Zhang & Wang, 2017 [22].

Most species of the family Eresidae are characterized by pronounced sexual dimorphism. In the genus *Eresus*, the known, described female specimens are stocky and robust in body build and generally black or blackish brown in color, while the males are often much smaller and have a red abdomen with black spots or, in some species, such as *E. elhennawyi*, black and cream-white patterns (Figure 1A–C) [16,17,18,19].

Kovács et al. (2015) report prosoma lengths of 2.6–4.2 (average 3.6) for *E. kollari*, 2.9–4.1 (average 3.6) for *Eresus sandaliatus* Martini & Goeze, 1778, 2.9–4.1 (average 3.4) for *Eresus hermani* Kovács, Prazsák, Vári & Gyurkovics, 2015, and 3.5–5.6 (average 4.6) for *Eresus moravicus* Rezác, 2008 [17]; these data concern the significantly smaller males.

Of the three species known and described from Morocco to date, *E. gharbi* is similar in habitus to the European species (Figure 1D–G), but its prosoma is almost completely black [17]. In male *E. gharbi*, the pars thoracica is heavily covered with red hairs. In this species, the second to IV legs are almost completely red, while among European species, *E. hermani* has a similar coloration on the legs, but the last segments of the II are black. The opisthosoma of *E. gharbi* is covered with red hairs and has four black spots in a similar distribution to European species.

The abdomen of European males is red and usually has four black, oval/circular spots, except for *E. sandaliatus* males, which have two larger spots followed by a pair of much smaller ones [5].

A species very similar in appearance to *E. kollari* (*Eresus tristis* Kroneberg, 1875) is known in Asia, in eastern Kazakhstan and the Xinjiang province of China, where they can be distinguished based on the micromorphology of the palpus [20]. Recently, *E. urus* Al-Yacoub & Zamani, 2025, described in Iraq, also resembles European species in appearance, but here the male’s pars cephalica is covered with grayish-white hairs and has a narrow white band running marginally on both sides (Figure 1H,I) [21]. *E. granosus* Simon, 1895, known from Asia, is similar in appearance to this species, although here the edge of the abdomen is black, and a line-like white stripe runs around the edge of the red area [22].

Iran appears to be a region rich in *Eresus* species. Following the description of *Eresus adaleari* Zamani & Szűts, 2020, from Iran, new species (*Eresus agrinus* Zamani & Szűts, 2025; *Eresus athanatoi* Zamani & Szűts, 2025; *Eresus marmoratus* Zamani & Szűts, 2025; *Eresus rezaci* Zamani & Szűts, 2025; *Eresus robin* Zamani & Szűts, 2025; *Eresus sparabara* Zamani & Szűts, 2025; and *Eresus surena* Zamani & Szűts, 2025) have been described in the Iranian region [13,18]. Of these species, four (*E. robin, E. agrinus, E. rezaci,* and *E. surena*) clearly exhibit ladybird spider morphology, while one (certain specimens of *E. athanatoi*) exhibits it to a lesser extent, with two black spots visible on their red-haired opisthosoma (Figure 1J–N) [13].

The appearance of *Eresus solitarius* Simon, 1873, described in the Iberian region, shows the morphology of the ladybird spider very similar to that of *E. sandaliatus*. The thoracic part of the cephalothorax is red, but the cephalic part is black in the *E. solitarius* [23,24]. *Eresus transcaucasicus* Zamani, Seropian, Zarikian, Bulbulashvili & Szűts, 2025, described in Armenia, has a similar appearance to the sandaliatus group. It resembles *E. kollari* and *E. hermani*. The cephalothorax is black, and the abdomen contains two large and one very small black spot on a red base at the dorsal surface (Figure 1O) [25].

After reviewing the available literature, we found no species among those described so far as exhibiting the morphology of the so-called ladybird spider whose pars cephalica was completely red, either in Europe, North Africa, or Asia. Our aim was to prove, based on characteristic morphological marks and genetic analysis, specimens found by us belong to a new species, spec. nov.

## 2. Materials and Methods

### 2.1. Samples

The here-investigated spiders were all collected individually and stored in 70% ethyl alcohol solution. The holotype of the collected spider has been deposited (id: HNHM Araneae-13777) in the Soil Zoological Collection (former Arachnoidea Collection) of the Department of Zoology, Hungarian Natural History Museum (HNHM), Budapest (Eszter Lazányi). The paratype of the collected spider was deposited (ID: 002-2025) at the University of Veterinary Medicine Budapest, Department of Exotic Animal, Wildlife, Fish, and Honeybee Medicine (Head of Department: Dr. János Gál).

### 2.2. Morphological Examination

We examined the habitus of two male specimens of the species described here, as well as the stereomicroscopic and scanning electron microscopic morphology and genetics of the paratype palpus. As part of the morphological examination, we measured the length of the prosoma in the holotype and paratype, which we give in mm. Habitus and palpus photographs were taken using a Keyence VHX-5000 (Keyence International, Mechelen, Belgium) digital microscope.

### 2.3. SEM

Scanning electron micrographs were made in a low vacuum using a Hitachi FlexSEM 1000 II (Hitachi High-Tech Corporation, Tokyo, Japan) scanning electron microscope at the Plant Protection Institute of the HUN-REN Centre for Agricultural Research (Martonvásár, Hungary). Objects were coated with 20 nm of gold before examination.

### 2.4. Genetic Examination

Nucleic acid extraction: The right-side II, III, and IV legs of the spider paratype were homogenized in PBS with a stainless-steel bead for 15 min at 50 Hz using a TissueLyzer LT (Qiagen, Hilden, Germany) machine. The homogenate was centrifuged at 10,000× *g* for 5 min. Total DNA was extracted directly from the supernatant of the tissue lysate using the DNeasy Blood & Tissue Kit (Qiagen, Hilden, Germany) according to the manufacturer’s instructions. The DNA library was prepared with the Illumina Nextera XT DNA Library Preparation Kit and the Nextera XT Index Kit v2 Set C (Illumina, San Diego, CA, USA) [26]. The library pool was loaded onto an iSeq 100 i1 Reagent cartridge and was sequenced on an iSeq 100 sequencing platform (Illumina, San Diego, CA, USA).

### 2.5. Software

The 2 × 150 bp paired-end raw reads were mapped with the built-in mapper of the Geneious Prime v.2025.1.2 software (Biomatters, Auckland, New Zealand) to mitochondrial cytochrome c oxidase subunit I (cox1, or COI) sequences of Eresidae species deposited in the GenBank. Accession numbers of references, where available, are included in the generated phylogenetic tree. Raw sequence data were deposited in the SRA database with acc. no. PRJNA1313631. The resulting contigs were checked and extended manually, and another revision was carried out with remapping of the raw reads to the resulting contigs. The GenBank accession number of the assembled novel sequence is PX111731. Sequences were edited and aligned with the MUSCLE algorithm of the AliView software v1.28 [27]. Pairwise distance values were calculated with the Kimura 2-parameter (nucleotide sequences, nt) and p-distance model (nt and amino acid sequences, aa) of the MEGA11 software [28].

Both unrooted and rooted maximum-likelihood phylogenetic trees of 656 nt long cox1 sequences were generated with the TN93+G+I model, using the best fit model option of the PhyML 3.0 online tool, with 1000 transfer bootstrap replicates. The rooted maximum-likelihood phylogenetic tree of 218 aa long cox1 nt sequences was built with similar settings, but with the Q.pfam+R+F model. The tree was visualized with the MEGA 11 software [28,29].

Species delimitations were performed using various methods with default parameters: ASAP (Jukes–Cantor, JC69, and Kimura-2P, K80) and ABGD (X value was set to 1.0) via iTaxoTools 0.1. The results were saved in SPART format and compared using LIMES (iTaxoTools 0.1) [30].

## 3. Results

*Eresus rubrocephalus* sp. n., Zoobank LSID: urn:lsid:zoobank.org:pub:EF18B350-ED79-46D7-85F6-2DB2F2A1973A.

### 3.1. Material Examined

#### 3.1.1. Holotype

One male—Morocco, near Sidi Allal El Bahraoui (34°05′21.06″ N and 6°23′29.63″ W), singled, 2021-06-10, name of the collector: Richárd Bagyó (collection number: HNHM Araneae-13777).

#### 3.1.2. Paratype

One male—Morocco, near Sidi Allal El Bahraoui (34°05′21.06″ N and 6°23′29.63″ W), singled, 2021-10-06, name of the collector: Richárd Bagyó (collection number: 002-2025, Araneae).

### 3.2. Etymology

Unlike the previously known coloration of the *Eresus* genus (sandaliatus group), the prosoma of the examined specimens is uniformly red in color; see “*rubrocephalus*”.

### 3.3. Diagnosis

The habitus of males was like the European and some Asiatic *Eresus* sp. of the same sex.

**Figure 2:**
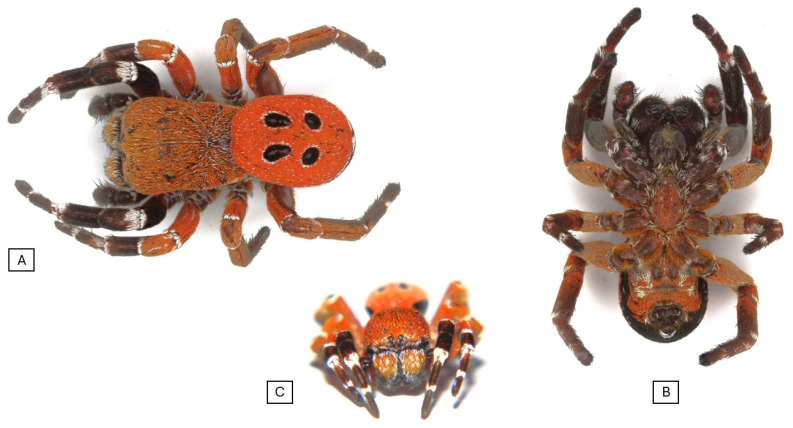
Habitus photo of the *Eresus rubrocephalus* sp. n. (**A**) Dorsal view, (**B**) ventral view, and (**C**) frontal view.

**Figure 3:**
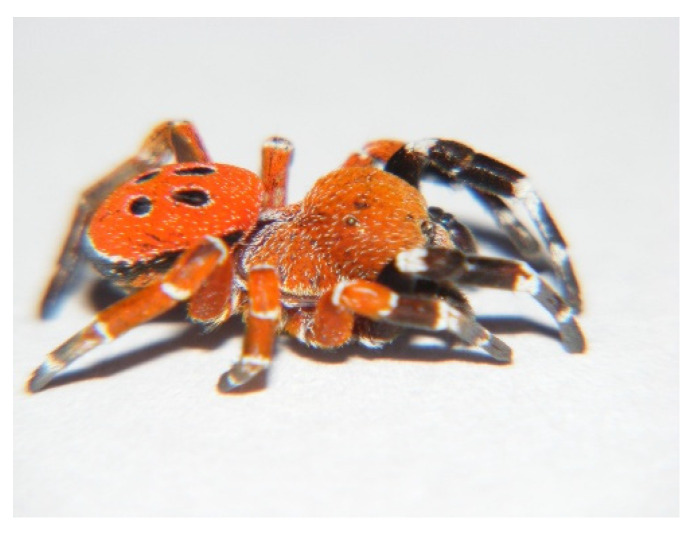
Prosoma shape in *Eresus rubrocephalus* sp. n. holotype (lateral view).

**Figure 4:**
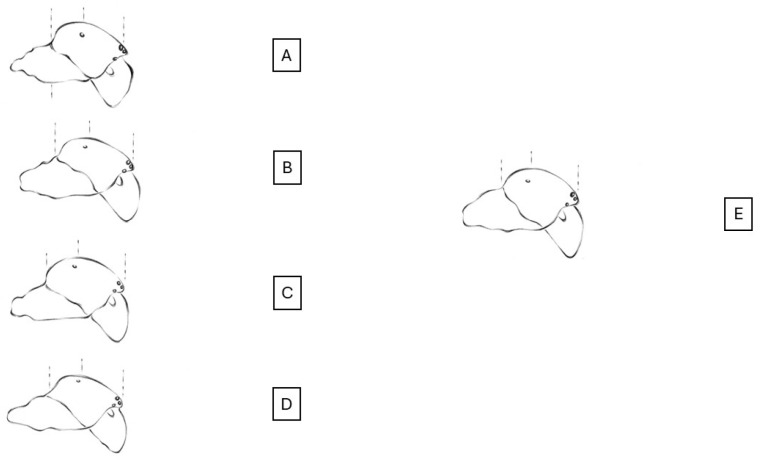
Outline of male prosoma of European *Eresus* spp. and related species with the *E. rubrocephalus* sp. n. (**A**) *E. hermani*, (**B**) *E. moravicus*, (**C**) *E. kollari*, (**D**) *E. sandaliatus*, and (**E**) *E. rubrocephalus* sp. n. [17] (drawn by Anna Ditta Dénes).

In our specimens, as in the *Eresus* sp. males, the clypeal hood forms a clearly acute angle, and the cephalic region of the prosoma does not overhang the thoracic region posteriorly (Figure 2, Figure 3 and Figure 4) [31]. Our two male specimens differ from all known species males in that the carapace dorsally and ventrally, as well as the chelicerae, are most uniformly carmine red (Figure 4). In the previously known ladybird spider species (*E. gharbi*, *E. solitarius*, *E. moravicus*, *E. sandaliatus*, *E. transcaucasicus*, and *E. agrinus*), pars thoracica is predominantly black compared to the species we described (Figure 1C–M,O) [13,15,17,18,21,22,25].

The abdomen is carmine red with oval-shaped spots on the dorsal part (Figure 2), but the first pair of dots shows a raindrop shape. These spots are circular or oval in the well-known European and similarly Moroccan species (Figure 1C–M). In the case of *E. hermani*, there is a narrow black stripe on the side of the abdomen (Figure 1G), which is also visible in *E. urus*, but in this species, it is extremely thin (Figure 1H) [17,21]. This is absent from the species we describe.

**Figure 5:**
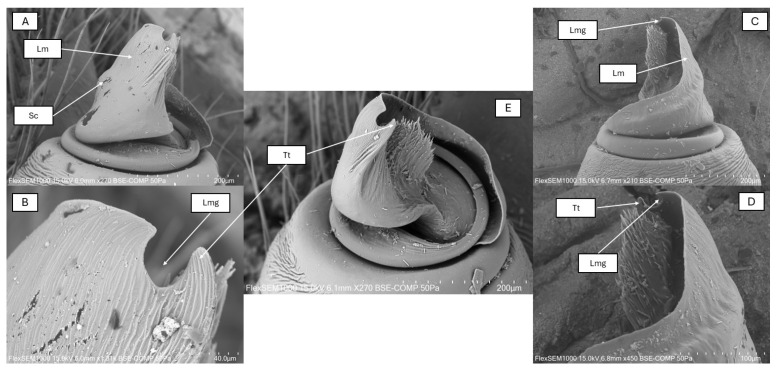
Electron microscopic image of palpus of *Eresus rubrocephalus* sp. n. (**A**,**B**) Retrolateral view, (**C**,**D**) prolateral view, and (**E**) ventral view. Lm: lamella, Lmg: lamellar groove, Tt: terminal tooth, Sc: shoulder of the conductor.

The palpus conductor plate (Lm) runs in a semicircle with a steep incline arc, with a significantly bulging, U-shaped groove (Lmg) at its apical end (Figure 5). This steep incline edge of the conductor plate continues after 130° of bending with a wide, flat, straight section. After this platform there is a U-shaped incision, which, unlike in known species, forms more than a semicircular shape, reaching almost three-quarters of a circle. This groove is very similar to that of *E. sandaliatus* and *E. moravicus*, but differently from the semicircular shape in *E. sandaliatus* and *E. moravicus,* this incision is much deeper in the newly described species, overreaching the semicircular rate and reaching three-quarters of a circle shape. In this species, unlike known species on the conductor plate, run two characteristic transversal wrinkles. Furthermore, this newly described species has on the lateral side of the conductor plate a blunt crest with a parallel shallow furrow, both running spirally to the apical part. In the species *E. rubrocephalus* sp. n., the terminal tooth (Tt) is pointed but not sharp-edged and curved.

### 3.4. Description

#### 3.4.1. Male

Prosoma. It has a length of 5.68–8.95 (mean 7.31, *n* = 2). The rate of pars cephalica and pars thoracica of the prosoma is like other *Eresus* spp. The pars cephalica is slightly wider than the pars thoracica. The shape of the prosoma corresponds to that of the “sandaliatus group”, but in the case of *Eresus rubrocephalus* sp. n., the pars cephalica is more arched, and the pars thoracica is also more convex (Figure 2 and Figure 3) [31].

The carapace is covered with mostly uniform carmine red hairs (Figure 2), with a few white hairs also visible, which are more numerous in the pars thoracica area. The lower part of the pars thoracica, the sternum, also has scattered red hairs, interspersed with a few solitary white hairs.

Chelicerae. Paturons and clypeus are also covered with carmine red hairs, with a few solitary white hairs (Figure 2).

Legs. One (I) pair of legs is black with a white band at the joints. Trochanters, femora, and patellae of II legs are red; distal edges of femora, patellae, tibia, and metatarsi have narrow, white stripes. Dorsally, the III and IV pairs of legs are uniformly red in color, with a white band at the joints (Figure 2).

Opisthosoma. The dorsal side of the abdomen is covered with carmine red hairs (Figure 2 and Figure 3). Two pairs of black spots are visible on the midline; the first pair of spots is drop-shaped, and the second is slightly oval. The spots are surrounded by a narrow, line-like white border. The ventral side of the opisthosoma is black with the exception of the red-haired area from the branchial opercula to the spinnerets.

**Figure 6:**
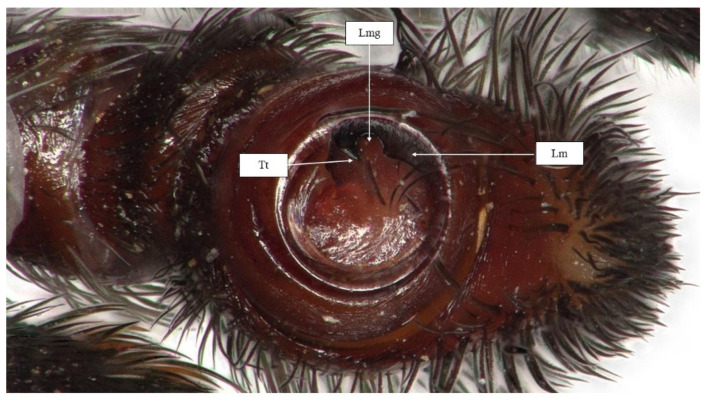
Palpus photo of *Eresus rubrocephalus* sp. n. (holotype). Lm: lamella; Lmg: lamellar groove; Tt: terminal tooth.

Palps. Based on the light microscopic image of the palps of the examined specimen (holotype), a deep, hollow groove widening at the bottom, characteristic of the *sandaliatus* group, can be seen on the conductor plate (Figure 6).

In the scanning electron microscope image, the conductor plate runs in a curved, semicircular arc. The plate is separated from the basal tubercle by a shallow indentation, after which it rises steeply, then with a 130-degree angle continues in a flat apical part with a U-shaped groove followed by a curved, not very sharp-edged terminal tooth. The groove’s depth is 30% larger than its width, instead of the semicircular shape reaching three-quarters of a circle. At the end of the conductor plate of *E. rubrocephalus* sp. n., slightly above the groove, there is a hook-like tooth that tapers at the end. From its base, in the species we describe, two shallow transverse wrinkles diagonally towards the embolus on the outer surface of the conductor plate smooth out at about half the height of the plate, accompanied by numerous very shallow wrinkles. Fine wrinkling is visible on the lateral surface of the terminal tooth. In the *E. rubrocephalus* sp. n., the conductor plate has a blunt crest running parallel with a shallow furrow spiraling upwards to the apical part. In the new species we have described, the conductor plate is slightly taller than it is wide (Figure 5). A deep gap running between the tegulum and the embolus can be observed, which is uniform in width and depth along its entire arc.

#### 3.4.2. Female

Female: unknown.

Distribution. Known from one locality—Morocco, northeast from Sidi Allal El Bahraoui.

Habitat. The two males were found in sparse undergrowth covered with herbaceous plants, in a grove of cork oak (*Quercus suber*) trees. The area has red sandy soil rich in iron compounds.

Phenology. According to our current knowledge, *Eresus rubrocephalus* sp. n. matures in June, and wandering males can be found at this season.

### 3.5. Genetic Examination

**Figure 7:**
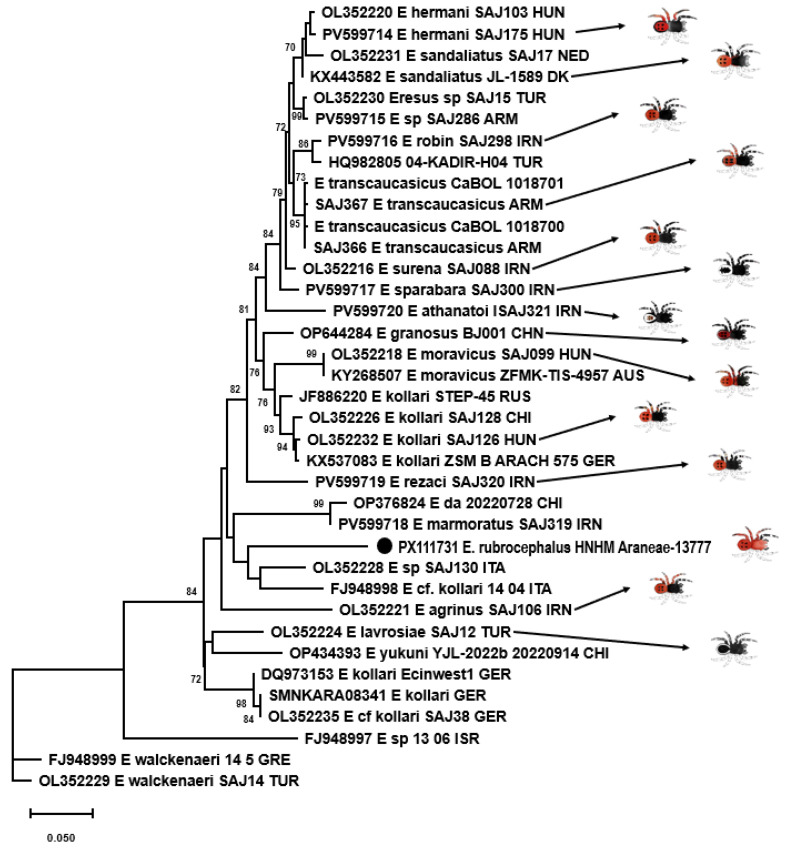
Unrooted maximum-likelihood phylogenetic tree of cox1 nucleotide sequences of Eresidae species. The tree was generated with the TN93+G+I model of the PhyML 3.0 software, with 1000 transfer bootstrap replicates. The sequence originating from the *Eresus rubrocephalus* sp. n. was labeled with a black circle.

**Figure 8:**
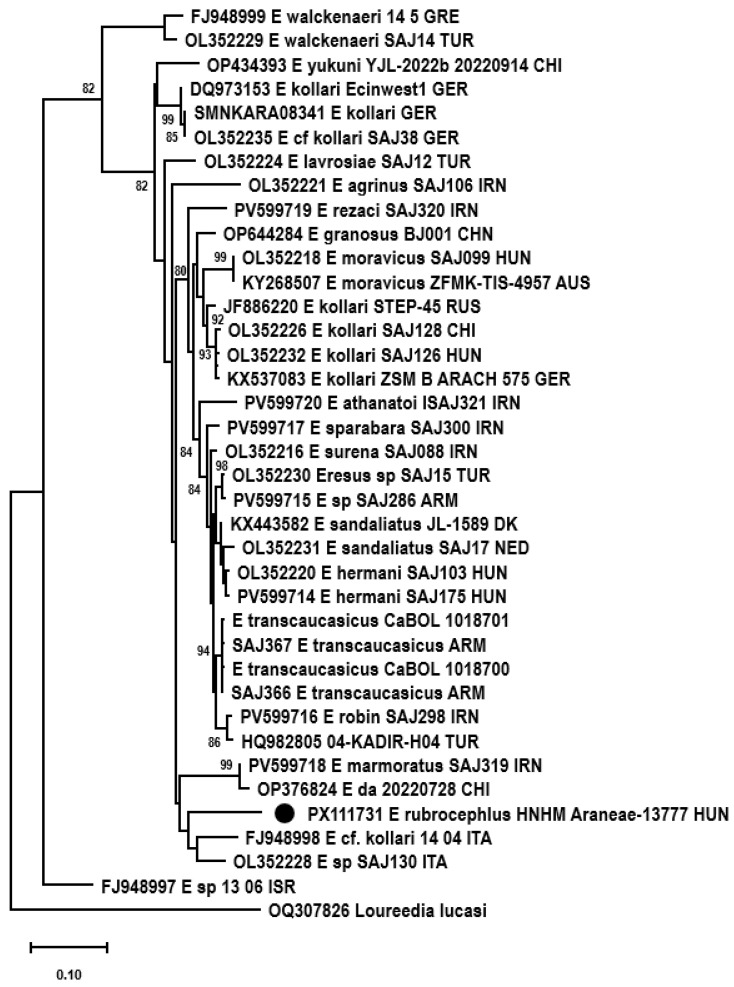
Maximum-likelihood phylogenetic tree of cox1 nucleotide sequences of Eresidae species, rooted to the cox1 nucleotide sequence of *Loureedia lucasi*. The tree was generated with the TN93+G+I model of the PhyML 3.0 software, with 1000 transfer bootstrap replicates. The sequence originating from the *Eresus rubrocephalus* sp. n. was labeled with a black circle.

**Figure 9:**
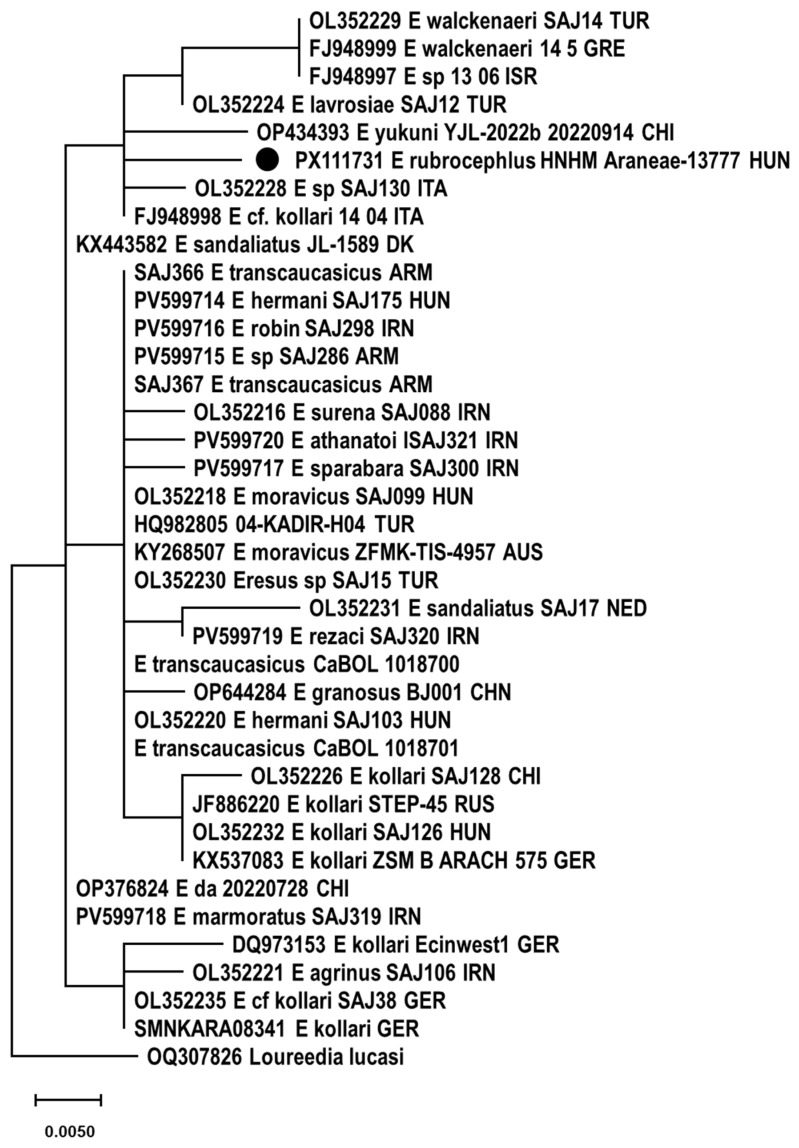
Maximum-likelihood phylogenetic tree of cox1 amino acid sequences of Eresidae species, rooted to the cox1 amino acid sequence of *Loureedia lucasi*. The tree was generated with the Q.pfam+R+F model of the PhyML 3.0 software, with 1000 transfer bootstrap replicates. The sequence originating from the *Eresus rubrocephalus* sp. n. was labeled with a black circle.

Altogether, 2448 sequence reads mapped to cox1 references with a mean coverage of 15 (0–619). At last, a 1233 nt long consensus sequence could be assembled. Phylogenetic analysis was performed using a 656-base-pair-long fragment for comparison with data in the literature. *E. rubrocephalus* sp. n. was located on a well-separated branch in the rooted and unrooted nt and aa sequence-based phylogenetic trees (Figure 7, Figure 8 and Figure 9). It was distantly related to *E.* cf. *cinnaberinus* ITA 14 04 and *Eresus* species ITA SAJ130 that, together with *E. agrinus* from Iran, were recognized as a separate lineage through haplotype and phylogenetic analysis [25].

Pairwise identities were calculated with p-distance and K2P models. The cox1 sequence of the *E. rubrocephalus* sp. n. represented ≥ 8.0% nt and ≥0.6% aa pairwise identity with the references, calculated with the p-distance model, while this value was ≥8.6% with the K2P model applied for nt sequences. Alignment of aa sequences revealed one unique aa substitution compared to other *Eresus* cox1 sequences.

**Figure 10:**
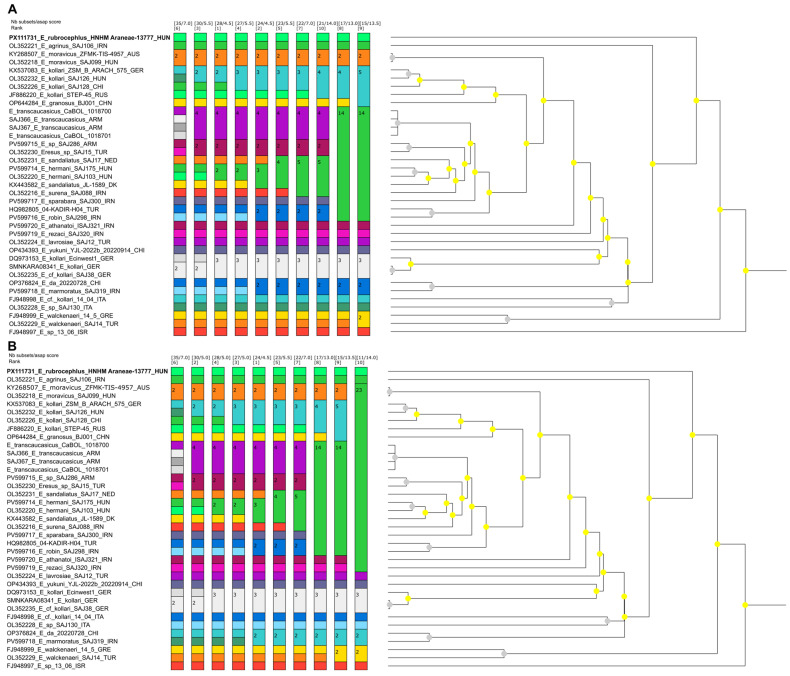
Species delimitation analyses of cox1 sequences of Eresidae species. The name of the novel species, *Eresus rubrocephalus* sp. n., is highlighted in bold. Panel (**A**): ASAP analysis with the Jukes–Cantor (JC69) substitution model. Panel (**B**): ASAP analysis with the Kimura-2P (K80) substitution model.

Species delimitation of certain groups of sequences changed dynamically depending on the scoring, which has also been pointed out before [13,25]. However, *E. rubrocephalus* sp. n. was represented as a distinct, single species in any comparisons (Figure 8, Table S1). This was found also for a few other, well-separated sequences in the phylogenetic tree, such as that of the closest relatives of *E. rubrocephalus* sp. n., the *E.* cf. *cinnaberinus* ITA 14 04 and *E.* species ITA SAJ130, or the *E. agrinus* and E_sp_13_06_ISR (Figure 7, Figure 8, Figure 9 and Figure 10). Overall, the results of the genetic analyses confirmed our assumption that a new species was established.

## 4. Discussion

Species of the ladybird spider morphological group are continuously described from Asia, the Middle East, the Mediterranean region, the Iberian Peninsula, North Africa, and Middle Europe [5,13,15,17,18,21,22,25]. Male spiders are characterized by an abdomen covered primarily with carmine red hairs, which have black spots visible on them. In the species described so far, the cephalothorax is almost black. In some species, including *E. gharbi*, *E. solitarius*, *E. moravicus, E. transcaucasicus*, *E. agrinus,* and *E. sandaliatus*, red hairs can be seen in the pars thoracica [15,17,24]. In our species (*E. rubrocephalus* sp. n.), the cephalothorax is entirely carmine red. This characteristic macroscopic appearance makes *E. rubrocephalus* sp. n. significantly distinct from the known ladybird spider species. The unique coloration of this species can be considered as a distinguishing mark.

Examining the palpus morphology, some differences were found also between *E. rubrocephalus* sp. n. and the known similar *Eresus* sp. One of these differences is the U-shaped groove, which is semicircular-shaped in *E. sandaliatus*, while it is three-quarters of a circle-shaped in *E. rubrocephalus* sp. n. The same groove is V-shaped in *E. kollari*, while in *E. moravicus* it is more rounded [17]. However, in the case of *E. elhennawyi*, known from Morocco, it is not a notch but a shallow indentation that breaks the arc of the conductor plate end [16]. In our species the terminal tooth is sharp-pointed and heavily curved, unlike in *E. kollari* and *E. sandaliatus*, where it is rather straight, and in *E. hermani* and *E. moravicus*, where it is strongly curved but wider, and in *E. hermani*, broad and blunt-ended [17]. In *E. adaleari*, this tooth is narrow but only slightly curved [18]. The width and height of the conductor plate are almost equal in *E. rubrocephalus*, slightly narrower than taller, while in *E. hermani* and *E. kollari* it is taller, in *E. moravicus* it is wider, and in *E. sandaliatus* it is almost as tall as it is wide [17].

The characteristics listed here clearly distinguish the *E. rubrocephalus* sp. n. from other *Eresus* species, but genetic evidence plays an important role in species demarcation. The availability of unique sequences of a species and the single locus-based species delimitation make genetic species assessment difficult [13,25,32,33]. Although the classification of species and species groups is uncertain in many cases within the genus, the phylogenetic and species delimitation analyses results demonstrate that *E. rubrocephalus* sp. n. forms a new species.

The description of *E. rubrocephalus* sp. n. and three other new species (*Eresus almaghrib*, *Eresus gharbi*, and *Eresus elhennawyi*) suggests the existence of a species formation and evolutionary hot spot in North Africa focusing on Morocco, similar to that revealed in the Middle East [13,14,15,16]. Deeper macro- and micromorphological investigation, in concert with genetic analysis, could greatly contribute to our understanding of the diversity and species development in the referred region.

## 5. Conclusions

The ladybird spider species, found in a cork oak tree (*Quercus suber*) forest in North Africa, Morocco, is strikingly different in appearance and coloration from the known species. According to the special external characteristics, its scientific name was composed from the Latin “ruber” (red) and the Greek “κεφάλι” (head) words and referred to as *E. rubrocephalus* sp. n. The morphological features and results of the cox1-based genetic analyses demonstrate that the here-characterized spider belongs to a novel species. As our study highlighted, the diversity of the *Eresus* genus justifies further and wider surveillance of variable geographical regions.

## Data Availability

The GenBank accession number of the assembled novel sequence is PX111731. Raw sequence data were deposited in the SRA database with an Acc. No. PRJNA1313631. The alignment (containing the novel sequence) used for genetic analyses is available in Figure S1.

## Supplementary Materials

The following supporting information can be downloaded at: https://www.mdpi.com/article/10.3390/ani15182707/s1. Table S1: Results of the species delimitation analyses. The analyses were performed using various methods with default parameters: ASAP (Jukes–Cantor, JC69, and Kimura-2P, K80) and ABGD (X value was set to 1.0) via iTaxoTools 0.1; Figure S1: Alignment of mitochondrial cytochrome c oxidase subunit I (cox1) sequences used for genetic analyses of *Eresus rubrocephalus* sp. n.

## Abbreviations

The following abbreviations are used in this manuscript:

Lm	lamella
Lmg	lamellar groove
Sc	shoulder of the conductor
Tt	terminal tooth

## References

[1] Canard A., Cruveillier M. (2016). Rectifications nomenclaturales á apporter au catalogue Mondial des araigées (1ére note). Rev. Arachnol..

[2] Geci D., Naumova M., Ibrahimi H., Grapci-Kotori L., Gashi A., Bilalli A., Musliu M. (2023). Contribution to spider fauna (Arachnida: Araneae) from Bjeshket e Nemuna Mountains (Kosovo). Nat. Croat..

[3] Thaler K., Knoflach B. (2002). Zur Fantastik der Spinnen (Araneae) von Österreich: Atypidae, Haplogynae, Eresidae, Zodariidae, Mimetidae. Linz. Biol. Beitr..

[4] El-Hannawy H.K. (2004). Review of spiders of genus *Eresus* in Egypt (Araneida: Eresidae). Serket.

[5] Rezác M., Pekár S., Johannsen J. (2008). Taxonomic reniew and phylogenetic analysis of central European *Eresus* species (Araneae: Eresidae). Zool. Scr..

[6] Wang J.F. (1994). Two species of spiders of the genus *Eresus* from China (Araneae: Eresidae). J. Hebei Norm. Univ.

[7] Breitling E., Bauer T., Schafer M., Morano E., Barrientos J.A., Blick T. (2016). Phantom spider 2: More notes on dubious spider species from Europe. Arachnol. Mitt..

[8] Naumova M., Deltshev C. (2021). New faunistic and taxonomic notes on the haplogyne and cribellate spiders (Araneae: Dictynidae, Dysderidae, Eresidae, Filistatidae, Sicariidae) from three Balkan countries. Acta Zool. Acad. Sci. Hung..

[9] Yanul V., Terekhova V., Polchaninova N. (2022). New data on the rare spider species (Arachnida, Araneae) from Kyiv region (Ukraine). Zoodiversity.

[10] Hoyas J., Ferrnández M.Á. (2023). Aranas (Araneae) de la Reserva fluvial del Rio Guadyerbas, Toledo centro de Espana. Rev. Iber. Arachnol..

[11] Kara C., Demir H., Seyyar O. (2025). New locality record of *Eresus lavrosii* Mcheidze, 1997 (Araneae: Eresidae) in Antolia. Serket.

[12] Seropian A., Bulbulashvili N., Makharadze G., Kovács G. (2025). From burrows to spotlight: First description of the female of *Eresus lavrosii* Mcheidte, 1997 (Araneae, Eresidae), with notes ont he natural history. Caucasiana.

[13] Zamani A., Seropian A., Zarikian N., Bulbulashvili N., Szűts T. (2025). Different, but still the same: Integrative taxonomy confirms a new species of *Eresus* Walckenaer, 1805 (Araneae, Eresidae) from the South Caucasus. ZooKeys.

[14] World Spider Catalog. Version 26. https://wsc.nmbe.ch/.

[15] Lecigne S., Moutaouakil S., Lips J. (2025). Contribution to the knowledge of the spider fauna of Morocco (Arachnida: Araneae)–Second note, on new species and new records from caves and miscellaneous terrestrial ecosystems. J. Belg. Arachnol. Soc..

[16] Rezác M., Vanek O., Strestík V. (2023). *Eresus elhennawyi* sp. n. (Araneae: Eresidae), a new velvet spider mimicking mutilid wasps from north-western Africa. Serket.

[17] Kovács G., Prazsák I., Eichardt J., Vári G., Gyurkovics H. (2015). A new ladybird spider from Hungary (Araneae, Eresidae). ZooKeys.

[18] Zamani A., Altin C., Szűts T. (2020). A black sheep in *Eresus* (Araneae: Eresidae): Taxonomic notes on the ladybird spiders of Iran and Turkey, with a new species. Zootaxa.

[19] Popovici G., Iorgu E.I. (2022). First record of *Eresus moravicus* Rezác, 2008 (Araneae: Eresidae) from Romania. Arachnology.

[20] Marusik Y.M., Azarkina G.N. (2020). Who is *Eresus tristis* Kroneberg, 1875 (Aranei: Eresidae)?. Arthropoda Sel..

[21] Al-Yacoub G.A.A., Al-Budeiri A.S.M., Zamani A. (2025). A new species of *Eresus* Walckenaer, 1805 (Araneae: Eresidae) from Iraq. Arachnology.

[22] Zhang Z.S., Wang L.Y. (2017). Chinese Spiders Illustrated.

[23] Simon E. (1873). Aranéides nouveaux ou peu connus du midi de l’Europe (2e mémorie). Mémories Soc. R. Sci. Liége.

[24] Breitling R. (2020). South European spiders from the Duffey collection in Manchester Museum (Arachnida: Araneae). Arachnology.

[25] Zamani A., Szabó K., Szűts T. (2025). Persian treasures: Integrative tanonomy reveals high species’ diversity of ladybird spiders (Araneae: Eresidae: *Eresus*). Zool. J. Linn. Soc..

[26] Fehér E., Kaszab E., Bali K., Hoitsy M., Sós E., Bányai K. (2022). Novel Circoviruses from Birds Share Common Evolutionary Roots with Fish Origin Circoviruses. Life.

[27] Larsson A. (2014). A fast and lightweight alignment viewer and editor for large datasets. Bioinformatics.

[28] Tamura K., Stecher G., Kumar S. (2021). Molecular Evolutionary Genetics Analysis Version 11. Mol. Biol. Evol..

[29] Guindon S., Dufayard J.F., Lefort V., Anisimova M., Hordijk W., Gascuel O. (2010). New Algorithms and Methods to Estimate Maximum-Likelihood Phylogenies: Assessing the Performance of PhyML 3.0. Syst. Biol..

[30] Vences M., Miralles A., Brouillet S., Ducasse J., Fedosov A., Kharchev V., Kumari S., Patmanidis S., Puillandre N., Scherz M.D. (2021). iTaxoTools 0.1: Kickstarting a specimen-based software toolkit for taxonomists. Megataxa.

[31] Miller J.A., Griswold C.E., Scharff N., Rezác M., Szűcs T., Marhabaie M. (2012). The velvet spiders: An atlas of the Eresidae (Arachnida, Araneae). ZooKeys.

[32] Lin Y., Li S., Zhao X., Chen Z., Chen H. (2022). Two new *Eresus* species (Araneae, Eresidae) from Xinjiang, China. Biodivers. Data J..

[33] Robinson E.A., Blagoev G.A., Hebert P.D.N., Adamowicz S.J. (2009). Prospects for using DNA barcoding to identify spiders in species-rich genera. ZooKeys.

